# Exploring the Role of Early Career Medical Professionals From a Digital-Oriented University in Germany in Promoting Digital Health in Professional Settings: Qualitative Interview Study

**DOI:** 10.2196/86107

**Published:** 2026-06-24

**Authors:** Christopher Koch, Jan P Ehlers, Julia Nitsche, Theresa Sophie Busse

**Affiliations:** 1 Faculty of Health, Witten/Herdecke University, Alfred-Herrhausen-Straße 50, 58455 Witten, Witten, Germany, 49 2302 926 708; 2 Department of Didactics and Educational Research in Health Science, Faculty of Health, Witten/Herdecke University, Witten, Germany

**Keywords:** digital skills, digitalization, health care, medical education, medical graduates

## Abstract

**Background:**

To address care delivery gaps, the health care system must embrace innovative digital solutions. Additionally, the rising integration of digitalization as a topic into medical education is providing students with broader opportunities to engage with digitalization overall. As digital health becomes an increasingly integral component of medical education and health care practice, digitally affine early career medical professionals constitute a vital resource for advancing digitalization within the health care sector.

**Objective:**

This study examines how early career medical professionals from Witten/Herdecke University in Germany, with various courses focusing on digitalization, acquire digital knowledge, apply it across diverse practical contexts—ranging from start-ups and corporate environments to traditional clinical settings—and how they contribute to the advancement of digitalization within the entire health care sector.

**Methods:**

Using a qualitative approach, 19 interviews were conducted with early career medical professionals who graduated in the last 15 years at Witten/Herdecke University. Subsequently, the interviews were transcribed and analyzed using a deductive-inductive approach.

**Results:**

The findings reveal that medical graduates often acquire digital skills through intensive self-study and learning on the job, integrating them in various ways into their professional lives. Moreover, while graduates recognize their high potential to make their own contribution to the advancement of digitalization, they also face significant barriers such as knowledge gaps, limited resources, and complex regulations, which hinder their ability to contribute to digitalization in a variety of professional settings. Medical graduates report that they face a pressing need for enhanced knowledge access, improved institutional frameworks, and supportive policy measures to maximize their potential in advancing digitalization initiatives.

**Conclusions:**

Recent medical graduates represent an underused resource for health care digitalization. Unlocking this potential requires coordinated action across medical education, health care institutions, and policymaking to create appropriate conditions for graduates to actively drive digitalization.

## Introduction

### Background

The entire health care system in Germany is under large pressure to solve existing care delivery gaps and to maintain an appropriate and patient-oriented health care infrastructure [1,2]. Already today, health care providers and patients are confronted with many emergent changes and obstacles, preventing optimal medical care. In this context, one possible approach is the increased integration of digital solutions into the whole health care industry [3,4]. The provision of health care and well-being via technology itself is referred to by the umbrella term “digital health,” including various forms [5]. In general, 3 different types of digital offerings can be distinguished: the lowest level is “digitization,” where analog components are converted to digital data as an earlier technical process [6]. Followed by “digitalization” referring to “the integration of digital technologies into public health operations” [6], which affects all health care processes in a public health perspective. Digitalization additionally refers to the use of digital technologies to optimize business processes and value creation [6]. This is clearly distinct from digital transformation as a multifaceted process including a change in the culture and form of health care service delivery as well as an extension beyond the health care sector [6].

### Digitalization in Health Care

Therefore, as in many sectors, digitalization has also emerged as a pivotal strategy in health care [3,7,8], addressing care delivery gaps and offering innovative solutions to enhance the efficiency, quality, and accessibility of health care services [9]. Thereby, many stakeholders can benefit from digital opportunities [10,11]. Patients can use digital services to develop a deeper understanding of their illnesses, to save travel and waiting time by moving medical consultations to the digital space, or to integrate innovative treatment methods in the recovery process. Furthermore, digitalization enables patients to become an active and preventive part of their own care [10,12,13]. Additionally, medical practitioners can use digital services to reduce administrative work, to improve diagnostic quality, or to foster exchange with other health care institutions [10,14,15]. But also, health insurance companies can benefit from an overall efficiency potential, that is, through the reduction of double treatments or new emerging marketing opportunities [16]. To improve the actual practical standards in Germany, health care organizations must find ways to integrate more digital technologies—that is, the meanwhile better-known electronic patient record, the electronic prescription, the telematics infrastructure messenger, or telemedicine—into daily practice [17,18]. To realize this, health care institutions must solve different challenges; that is, they need to overcome institutional resistance or integrate digital approaches that align with data security and legal constraints [19-21]. Overall, there is further potential for improvement in the integration of technology and the design of pathways—especially regarding emerging technologies such as artificial intelligence (AI) [22-25]. Therefore, the health care sector must seek ways to integrate the full potential of digitalization into practice, while existing challenges may continue to slow down progress. Recent advances in AI and the growing prevalence of online misinformation increase the need for digital health literacy. Being able to evaluate the credibility of digital health information and services is essential to distinguish high-quality, evidence-based offers from misleading or potentially harmful content [26,27]. One way of supporting the digital advancement within the health care sector could be the consideration of early career medical professionals [28,29].

### Early Career Medical Professionals and Digitalization

Looking at their individual imprints, these early career medical professionals have grown up in the digital age and have experienced the emergence of digitalization since their early childhood [30-32]. In doing so, they have learned to constantly adapt to digital innovations and to use them in daily life [33]. In this context, they are particularly open to digital solutions and can handle computer-based technologies in an appropriate way [33,34]. Consequently, these people already have a pronounced digital affinity [35]. Also, during their studies, those medical students want to understand the benefits of new digital apps, making them ready to shape their professional environment [28,29].

At the same time, overall, digital literacy is becoming one of the key skills on the job market [36]. These generations will also occupy a large part of the labor market in the coming years and thus shape the entire health care industry [37-39]. Consequently, after graduation, these cohorts can become pioneers in transferring digital knowledge, that is, from academia or their private learning to the health care practice [40,41].

However, extensive private use of digital media does not automatically ensure that students integrate these technologies into their studies effectively, highlighting the need for clear education training strategies [42-44]. Consequently, to make an appropriate practice transfer come true, universities must educate students based on their digital needs and set the right framework by promoting appropriate and supporting digital content [45,46].

### Digitalization in Medical Studies

At least since the COVID-19 pandemic, increased digitalization is also affecting the study experience of medical students [47-49]. In recent years, more and more medical content has been taught in or transformed into digital formats [43,50,51]. Furthermore, the topic of digital health is also increasingly being integrated into the elective areas of various degree programs, showing the importance of digitalization in practice and consideration in medical education [52,53]. Thus, the medical curriculum is evolving, aiming to develop future-oriented digital skills and competencies [54]. In this context, medical lecturers as well as students classify the thematization of digitalization in study programs as an important part of modern medical education [55,56]. Nevertheless, as the National Competence-Based Learning Objectives Catalogue for Medicine in Germany has decided to allocate only a limited amount of time to digital content within the medical curriculum, many universities still consider a few digital medical topics throughout their studies, deriving an existing improvement potential in the full medical education market [57-59]. Regardless of the digitalization of study programs, students are also looking for new ways to optimize their studies with digital offerings, primarily focusing on innovative and digital resources to enhance learning outcomes and performance evaluation [60-62]. Here, digital learning opportunities give students an additional way to acquire and stabilize knowledge within their medical studies [63]. In addition to that, the student’s environment also becomes more digital, as universities are digitizing their processes and structures [64,65]. All these developments together show that medical students are confronted with an increased number of digital elements in their day-to-day life while becoming a professional physician, highlighting at the same time the improvement potential by this cohort.

### Research Question

It becomes clear that today’s medical students are experiencing an increased digitalization in health care, which will shape their professional future. Based on their personal experiences, they are probably open to digital innovations and new technologies. Exactly matching, increasing digitalization also offers an opportunity to improve the entire health care system and to solve existing care delivery gaps. Therefore, health care actors must find ways to better implement useful digital solutions into practice and discover problematic solutions or hurdles. As few existing studies have investigated the potential of early career medical professionals in shaping digitalization in health care practice, this study tries to connect these 2 strands and focuses on how, when, and why early career medical professionals can (not) transfer digital skills and knowledge into their professions, improving the practical standard—focusing on the perspective of this target group. Therefore, this study investigates early career medical professionals to understand the potential practical transfer, deriving implications for medical education and practice. Consequently, the research question is “How can early career medical professionals contribute to advance digitalization and digital transformation in practice?”

To answer this research question in full, 3 more subresearch questions were formulated:

- In which contexts do medical students learn digital skills, and how are these applied in practice?
- What potential do early career medical professionals have to contribute to advancing digitalization and digital transformation?
- What challenges does this target group face, and what possible solutions are there?

## Methods

### Ethical Considerations

This study, as well as its ethical and practical implications, has been approved by the ethics committee of Witten/Herdecke University (UWH), Faculty of Medicine (application number S-254/2024). All participants provided informed consent. Data were anonymized for the presentation in this paper. Participants did not receive compensation.

### Reporting Guideline

The COREQ (Consolidated Criteria for Reporting Qualitative Research) checklist [66] was applied and can be found in Checklist 1.

### Research Purpose

This qualitative study serves to provide insights into the practice transfer of digital skills by early career medical professionals. Thus, the research team wants to understand how and in which context early career medical professionals learn and apply digital skills, contributing to the advancement of digitalization in the health care sector. This research tries to contribute to this ambiguous problem by preparing fundamental content, which needs to be tackled with explorative research [67]. To gain these specific insights, a qualitative approach was adopted. Therefore, data were primarily collected for this specific research question by conducting exploratory interviews [68]. This research approach was deliberately chosen because qualitative interviews enable a deeper exploration of participants’ experiences and further data interpretation. Furthermore, as the research team pursues a mixed methods approach, the results will serve as the basis for an upcoming quantitative study.

### Sample and Recruitment

The participating interview partners were selected based on the following four criteria:

1. Only medical graduates of UWH as early career medical professionals were considered, as various optional courses on the basics of digitalization and digital health are offered at this university [69,70], and best practices should be identified.
2. Early career medical professionals from the 2 medical specialties offered at UWH were included: human medicine and dentistry.
3. Only early career medical professionals who have encountered digitalization in their studies or professional work in any form were considered (self-disclosure).
4. As the study focused on the role of early career medical professionals, only individuals who had graduated within the past 15 years were included. This broad time span was selected to encompass both individuals at the beginning of their careers and those with at least some distance to reflect on their experiences.

Participants were invited to take part in the study via multiple channels, including face-to-face meetings, telephone interviews, email, and the social media platform LinkedIn (LinkedIn Corporation). The participants were recruited from September 23, followed by a smooth transition to the interview phase. In this context, participants were contacted and interviewed until theoretical saturation was reached, meaning that further interviews no longer provided relevant new insights.

### Interview Guide

The interview guide can be found in the Multimedia Appendix 1.

To ensure comparability across the interviews, a semistructured interview guide was developed. In this context, the SPSS method (Sammeln, Prüfen, Sortieren, Strukturieren [German]; collecting, checking, sorting, subsuming [English]) was used for collecting, checking, sorting, and subsuming the questions within the research team [71]. Furthermore, the existing literature was considered while developing the guide. In sum, this allowed us to explore 3 key aspects in depth: learning digital skills, practical perspective (current status, potential, and challenges), and overcoming challenges. Each topic was accompanied by main and potential follow-up questions, enabling the researchers to address up to 4 questions per category. At the beginning of each section, open questions were asked, which became more closed as the interview section progressed. In addition to that, a deliberate question formulation was integrated to avoid social desirability and to foster creative thinking [68,72]. During the interviews, participants were encouraged to speak freely and share their experiences. Overall, the guide served as a framework to structure the interviews, enabling possible adaptation to changing interview situations.

Prior to conducting the interviews, the practical applicability was successfully tested with a smaller pilot group (n=4), which led to further refinement before finalizing the questions. The interview recordings from the pilot were neither transcribed nor included in the final analysis.

### Data Collection

From October 1, 2024, to November 30, 2024, 19 interviews were conducted by CK either in person or in a digital space by Zoom (version 6.4.10; Zoom Communications Inc).

Before conducting the interviews, the participants were informed about the study’s objective, design, and recording procedures. In this context, complete anonymity was assured throughout the evaluation and publication processes. All participants subsequently provided their consent to participate under the outlined conditions. For their participation, the interview partner received no remuneration. After the interviews, CK transcribed the data according to Przyborski and Wohlrab-Sahr [73].

### Data Analysis

In the next step, the interviews were analyzed and coded using MAXQDA (version 24; VERBI Software), a software suitable for qualitative data analysis. This was done between December 1, 2024 and January 31, 2025.

A qualitative content analysis based on Kuckartz and Rädiker was conducted [74]. The analysis was initially carried out separately by CK and TSB for 2 interviews. They then met to discuss the interviews, and an initial coding system was developed inductively. CK continued to work with this coding system and refined it while analyzing the remaining interviews. Where there were questions regarding categorization, CK consulted TSB, and they reached a mutual consensus. Once CK had completed the analysis of all interviews, TSB, JN, and JPE reviewed the findings. This was followed by a joint meeting with CK, JN, JPE, and TSB to discuss and finalize the analysis and coding system. Any discrepancies were resolved jointly at this meeting.

The final category manual presents a detailed overview of the individual categories and subcategories. Here, the first 3 coding levels were reported, as these concisely summarize the most important findings. Furthermore, the reporting will focus exclusively on codes that are relevant to addressing the research question. All published quotes have been translated into the English language and have been adapted grammatically.

## Results

### Overview

In the following, the key findings are presented, structured by the final coding scheme. A total of 19 interviews were conducted, lasting between 20 and 48 minutes, with a total duration of 10.4 hours, equivalent to 624 minutes. On average, each interview lasted 31 minutes. The participants’ characteristics are presented in Table 1.

**Table 1.** Participants’ statistics.

Parameters	Values, n (%)
Participants	19 (100)
Sex	
Male	11 (58)
Female	8 (42)
Background	
Human Medicine	17 (89)
Dentistry	2 (11)
Position	
Physician	13 (68)
Entrepreneur	6 (32)
Age (years)	
Minimum	26
Maximum	40
Average	34
Interview duration (min)	
Minimum	21
Maximum	48
Average	33

Overall, a total of 1640 coded segments and 1957 distinct codes were obtained through the entire analysis. Here, 5 codes belong to the first order, 17 codes to the second order, and 58 codes to the third order. The code structure can be found in Table 2. In the following, these first-, second-, and third-level codes are described in more detail. The participants did not provide feedback on the research findings.

**Table 2.** Code structure.

First-order code	Second-order code	Third-order code
Occasion learning digital skills	Job	For concrete activities
		As a basic working method
	Private	Due to digital touchpoints
		Through self-study
	Study	Through teaching
		Through research projects as part of dissertations
		In self-study during exam preparation
Application purpose digital skills	Administrative	Digital office software
		Clinical programs
	Strategic	Foundations
		Business model development
		Process optimization
	Communicative	Training and further education of others
		Leadership and negotiation
Potentials of early career medical professionals in advancing digitalization	Organizational characteristics	Recognition
		Error acceptance
	Personality traits	Impartiality
		Motivation
		Load capacity
		Willingness to change
		Learning ability
		Pragmatism
		Naivety
	Professional competences	Affinity and intuition
		Knowledge
		Digital ideas and interest
		Positive professional attitude
		Study proximity
	Social skills	Willingness to compromise
		Critical perspective
Challenges of early career medical professionals in advancing digitalization	Organizational	Role in the organization
		Diverging points of view
		Hierarchy
		Inadequate structures and processes
		Lack of incentives
		Lack of technical implementation options
		Established analog functionality
	Personal	No motivation
		Lack of personality traits
		Lack of knowledge
	Subject-specific	Regulation
		Data protection
		Personal burden
		Lack of networking
		Lack of investment and infrastructure
		High cost pressure
Success factors and possible solutions	Education	School
		Study
		Further training
	Alignment of the organization	Positioning for digitalization
		Support from management and leadership
		Change management
		Thinking through the system and culture
		Providing space and infrastructure
		Building digitalization expertise
	Politics and the state	Provide infrastructure
		Reviewing regulation
		Optimize data protection

### Learning Digital Skills

#### Overview

Early career medical professionals report that they have enhanced their digitalization knowledge in various settings—particularly during their studies, in their professional roles, and in their personal lives.

#### Study

During their studies, early career medical professionals were introduced to digitalization through curriculum-based teaching, dissertation projects, and self-directed learning. However, digitalization is still relatively underrepresented in the medical curriculum—even at UWH. When addressed, it is typically limited to foundational courses within the selection area, offering just a broader overview of digitalization potential, digital apps, or basic technical tools.

I attended a series of lectures on digitalization in medicine, which focused on innovations and their potential applications in future medical practice.

[Interviewee No. 16, Human Medicine, Physician, Female]

Consequently, early career medical professionals receive limited formal preparation to increase their digital knowledge within their medical study programs. Furthermore, early career medical professionals report that they familiarize themselves with digital tools through intense self-studies, particularly during exam preparation. In addition to that, some respondents acquired their foundational digital skills through past high school activities.

Sometimes, digitalization is explored in medical research, especially during dissertation projects.

#### Job

In their professional roles, early career medical professionals develop digital skills through an inherently digital work environment and related collaboration models. As a result, they frequently gained experience with digital programs, including advanced AI-based tools, which they encounter as part of their daily practice. Throughout this process, early career medical professionals passively acquired new digital skills, shaped by the demands and technologies prevalent in their specific environments. Additionally, early career medical professionals had to acquire deeper digital skills to perform specific job-related tasks and activities, especially to solve emergent entrepreneurial challenges. Therefore, the required skills were often developed through a hands-on learning-by-doing-approach.

Learning digital skills often happens naturally when encountering them in daily work, especially when solving an immediate problem. It’s not something I actively sought out, thinking, “What could I learn about digitalization today?” Rather, it’s something that gradually becomes part of the workflow.

[Interviewee No. 18, Human Medicine, Physician, Female]

#### Private

In their private lives, early career medical professionals report that they have developed digital skills through constant confrontation with digitalization over the years. Regular interaction with digital technologies in everyday activities—often since childhood—has fostered an intuitive and ongoing engagement with a variety of digital elements. Thus, this passive yet consistent immersion has naturally shaped their digital competencies over time. Beyond this passive acquisition, many early career medical professionals have actively developed digital skills through various personal initiatives. Here, some graduate students learned coding or programming during their leisure time, which broadened their technical and digital understanding in depth, enabling them to take on special professional tasks in practice later. Driven by personal interest, some early career medical professionals also pursued self-study through courses and training programs, deepening their engagement with overlapping digital megatrends.

I have also acquired some digital skills in my personal life out of genuine interest. For instance, I am currently learning to code. For me, this skill that is particularly relevant for understanding the growing prominence and impact of AI.

[Interviewee No. 6, Dentistry, Co-Founder, Male]

### Application Purpose of Digital Skills

#### Overview

Early career medical professionals report that they have applied their digital skills in different ways—especially in administrative, strategic, or communicative paths. Consequently, there are different approaches to bringing their digital knowledge into practice and sharing it with others.

#### Administrative

An administrative application occurs when early career medical professionals leverage their digital skills to manage simple and existing tasks or processes. In their workplaces, most early career medical professionals use digital software, tools, or communication platforms that have already been integrated—both in everyday clinical practice and in office-related settings.

In the hospital setting, a significant amount of time is spent on computers due to the need to document all information using digital systems.

[Interviewee No. 19, Human Medicine, Physician, Female]

Due to their strong digital affinity, early career medical professionals report that they can intuitively understand and incorporate these tools into their daily routines. The overarching goal of these efforts from the early career medical professionals’ perspective is to save time and ultimately improve the quality of work by enhancing workflow and coordination within their professional settings. In addition to that, some early career medical professionals take on other roles regarding digitalization, that is, acting as key users for a digital app.

#### Strategic

A strategic application occurs when early career medical professionals harness their digital skills to creatively and entrepreneurially advance digitalization initiatives. Thus, some digitally affine early career medical professionals even enhance internal processes by introducing new software, communication tools, or workflows, that is, digital lean management. Furthermore, early career medical professionals have also engaged in various entrepreneurial ventures, either independently or in the respective organization they are working at. In this context, they pursue a variety of different initiatives, such as developing digital business models or founding digital health-tech start-ups to address emerging market opportunities. As an example, some early career medical professionals have developed digital knowledge tools, providing easy access to relevant medical knowledge. Ultimately, these creative endeavors have enabled early career medical professionals to generate entrepreneurial value while actively contributing to the overall advancement of digitalization.

I would say that even before AI, we digitized quite a lot here in the company and simply looked into the processes, both on improvement potential on the patient side and in the processes towards employees.

[Interviewee No. 11, Dentistry, Managing Director, Male]

#### Communicative

A communicative application occurs when early career medical professionals share their digital skills with others through various means. In this context, especially early career medical professionals in a clinical practice pass on their digital expertise by organizing training sessions and engaging in dialogues with colleagues, where they inform them about emerging digital opportunities and recommend best practices. Consequently, some early career medical professionals also serve as multipliers, facilitating the adoption and integration of digital solutions. Furthermore, early career medical professionals also apply their digital skills in different management and negotiation contexts, using their expertise to enhance final work results.

It’s also important to take other medical colleagues by the hand and say: Hey, watch out, do it like this. I would say that I'm very active in this respect, that I'm a bit of a multiplier

[Interviewee No. 8, Human Medicine, Physician, Male]

### Potential of Early Career Medical Professionals in Driving Digitalization Forward

#### Overview

Early career medical professionals possess personal attributes and professional conditions that enable them to advance digitalization. These encompass organizational characteristics, personality traits, professional skills, and social skills.

#### Organizational Characteristics

Organizational characteristics encompass the advantages that early career medical professionals derive from their specific roles within their respective organizations. Therefore, early career medical professionals perceive that their needs are increasingly recognized as important by their employers, particularly considering demographic shifts that make these young professionals a valuable future resource. Aware of their value, some early career medical professionals report that they actively advocate for digital offers and, in some cases, even consider leaving employers who fail to meet their digital expectations. Additionally, early career professionals perceive themselves to benefit from a form of entry-level protection, where mistakes are more readily accepted. Furthermore, their perceived risks of failure remain relatively low. As a result, young early career medical professionals possess considerable potential to advance digitalization through their unique starting position.

You always enjoy a kind of “puppy protection” at the beginning. So, if you make mistakes, everyone tries to help you, and mistakes tend to be tolerated. That applies typically to the first six months.

[Interviewee No. 4, Human Medicine, Physician, Male]

#### Personality Traits

Personality traits encompass the personal characteristics that many early career medical professionals possess, supporting their ability to drive digitalization forward. In this context, early career medical professionals report that they bring an unbiased perspective, allowing them to notice dusty aspects, challenge organizational blind spots, and approach longstanding problems in a different way of doing. In this context, early career medical professionals perceive themselves as maintaining an outsider’s perspective that enables them to tackle problems with their fresh insights.

A huge advantage that you should try to retain is that you usually look at old problems with a fresh eye and then you may notice certain problems, for example structures that are not running well.

[Interviewee No. 1, Human Medicine, Physician, Male]

Furthermore, their intrinsic motivation and genuine commitment to drive meaningful change in health care, combined with a strong enthusiasm for digitalization, position them—from their perspective—as key agents of digitalization. Moreover, early career medical professionals perceive themselves as highly resilient. In this context, many early career medical professionals report that, having not yet assumed family responsibilities, they face fewer constraints than established leaders within their organizations. This relative flexibility allows them to devote more time and energy to exploring additional topics, such as advancing digitalization. Moreover, they demonstrate a high willingness to implement new digital solutions, as they point out to perceive the high potential of digitalization for real practical improvements. On top of that, they report their adaptability, pragmatism, and a certain degree of naivety as further advantages helping them to contribute to the advancement of digitalization.

#### Professional Competencies

Professional competencies include the technical and subject-specific skills that many early career medical professionals possess, enabling them to advance digitalization. Therefore, early career medical professionals point out a strong affinity for and interest in digitalization, having mostly grown up in a digital environment where they learned to navigate through digital topics with ease. They possess foundational digital knowledge and, in some cases, also specialized expertise—especially in comparison to older generations. Their enthusiasm for digitalization extends beyond theory, as they describe themselves as actively identifying practical opportunities for digital improvements. Furthermore, early career medical professionals note that they approach data protection with a pragmatic mindset. Additionally, early career medical professionals indicate that their proximity to academic studies could allow them to be specifically trained in digitalization right before entering the workforce, enabling them to immediately apply what they have learned.

So, I would say that graduates today no longer must learn the digital language or being digital from scratch, but that for many it is internalized like a second mother language. And that they therefore feel much less of a barrier or fear of having to adapt or learn it.

[Interviewee No. 15, Human Medicine, Co-Founder, Female]

#### Social Skills

Professional competencies include the interpersonal skills that many early career medical professionals possess, enhancing their ability to drive digitalization forward. Here, early career medical professionals perceive themselves tending to question the status quo, seeking ways to improve or optimize existing digital structures. Consequently, early career medical professionals report that they often adopt a critical perspective, questioning whether things are truly effective or necessary. Additionally, they describe themselves as open to compromise, which enables them to collaboratively find solutions when implementing digital solutions.

In conclusion, I think it is simply important to remain critical. In other words, to always ask critical questions: What is the opportunity? What is the limit? Is it useful? And we can certainly contribute a lot to this because we have been brought up as critical minds in the broadest sense.

[Interviewee No. 18, Human Medicine, Physician, Female]

### Challenges for Early Career Medical Professionals in Advancing Digitalization

#### Overview

But early career medical professionals also face general and target group-specific challenges—especially organizational, personal, and subject-specific hurdles.

#### Organizational

Organizational challenges encompass all obstacles that arise within the organization itself. As an example, some early career medical professionals state that they face challenges in gaining recognition within their organizations at all—especially in clinical settings. In this context, they report that they have limited influence and that driving digitalization is not perceived as one of their core responsibilities—both by their employers and by themselves. Thus, their initial priority is to establish their role and to integrate into existing structures, a process further complicated by frequent transitions between different workplaces. Consequently, early in their careers, they often focus on fulfilling assigned tasks and hesitate to step into a shaping role, fearing mistakes or drawing too much attention. Diverging perspectives within the workforce present additional obstacles that early career medical professionals describe. While some professionals advocate for digitalization, others—particularly senior staff—remain committed to analog practices, exhibiting limited openness to change. Hierarchical dynamics further exacerbate this issue, as decision-making power over digital initiatives rests primarily with established professionals, who—from the perspective of early career medical professionals—often resist transformation to maintain existing structures.

Of course, we are fresh, young and over-committed when it comes to some things. But that is simply not heard. [...] People don't take us that seriously. We young people come and say we want digital, and then the older ones say, yes, good for you.

[Interviewee No. 19, Human Medicine, Physician, Female]

Furthermore, early career medical professionals report that organizational constraints overall also impede digital progress. In this context, limited personnel resources, inflexible institutional frameworks, and the inability to disrupt ongoing health care operations create barriers to a successful digital improvement—especially in clinical settings. Moreover, early career medical professionals report a lack of incentives to engage in digitalization efforts, as neither formal support nor tangible rewards are provided. Likewise, technical limitations prevent the seamless implementation of digital solutions. Additionally, the existing functionality of analog systems diminishes the perceived urgency for digital changes.

#### Personal

Personal challenges encompass all obstacles stemming from the individual characteristics and circumstances of early career medical professionals. Here, not all early career medical professionals are intrinsically motivated to drive digitalization. While some prioritize efficiently fulfilling their true core responsibilities, others find digitalization less engaging or are reluctant to invest additional effort, particularly when it requires extra work or overtime. Additionally, some early career medical professionals do not feel as digitally inclined. Furthermore, they identify certain personality traits they feel they lack, which hinder their ability to contribute to digitalization. In this context, some exhibit a strong aversion to risk, some a lack of confidence, and some a lack of entrepreneurial mindset. Furthermore, early career medical professionals highlighted several knowledge gaps that hinder the adoption of digital tools, such as limited technical expertise, limited digital knowledge, and difficulties in navigating organizational processes.

There are few who are really motivated, so the term work-life balance comes up very early on. And to be honest, I also did these projects in my free time in overtimes.

[Interviewee No. 7, Human Medicine, Managing Director, Male]

#### Subject-Specific

Professional challenges include all obstacles that are rooted in specific content-related issues. First, even at the beginning of their career, health care professionals report that they are still overburdened, leaving little time and energy to engage in shaping digitalization. This was reported only by employees in a clinical setting.

What prevents young physicians from doing this is the time aspect. So you are overloaded with an extremely large number of medical and non-medical tasks.

[Interviewee No. 1, Human Medicine, Physician, Male]

Second, early career medical professionals argue that implementing digital solutions also requires significant financial investments, which are often hard to justify in the cost-sensitive health care sector. Furthermore, many early career medical professionals face bureaucratic processes and an outdated infrastructure. Third, early career medical professionals report that strict regulations prevent digital progress—especially due to strict data protection regulations. On top of that, early career medical professionals report that limited interconnectivity among health care institutions further impedes digital progress, as the lack of standardized digital collaboration systems prevents seamless data exchange.

### Opportunities to Drive Digitalization Forward

#### Overview

Early career medical professionals point out potential solutions to solve existing challenges, focusing on enhanced education, improved practice conditions, and changed political regulations.

#### Education

The area of education encompasses potential education-oriented measures. In this context, early career medical professionals emphasized the need for more comprehensive digital education and continuous professional development—especially during their medical study programs and, in some cases, also during high school. They suggested that universities should not only provide foundational digital courses but also offer practical, hands-on experiences that reflect real-world challenges and best practices, preparing students to actively contribute to advancing digitalization in health care practice. This preparation should focus on the specific content of digitalization, but also on the organizational challenges they will face, such as resistance to change. Therefore, universities should provide medical students with strategies on how to navigate these as a recent alumnus effectively. Furthermore, early career medical professionals report that fostering a digital and entrepreneurial mindset is crucial for early career medical professionals to solve a variety of challenges.

Digital skills, applications and the courage to integrate this innovation into must also be trained during the study program.

[Interviewee No. 4, Human Medicine, Physician, Male]

Thus, teaching students to think critically about how digitalization can be integrated into health care and different business models is a critical success factor from early career medical professionals’ perspective. Furthermore, early career medical professionals state that continuous workplace training is also essential to keep professionals up to date with rapidly evolving digitalization.

An employer should send a clear signal to the outside world that it is willing and has interest in digital innovations. They should clearly say: We want to actively promote this.

[Interviewee No. 17, Human Medicine, Physician, Female]

#### Politics and the State

Additionally, from the participants’ perspective, policy reforms are crucial to reduce bureaucratic hurdles, streamline processes, and provide more flexibility for digital initiatives. Therefore, reforms should aim to balance data security with the freedom to push digitalization, allowing for a safe but agile development of digital solutions. Furthermore, the state should establish a robust infrastructural framework to support the development and implementation of digital initiatives.

This is very top-down. That’s typical with the EU directives, they always come from the top and are not coordinated with the market. And I would like to see these hurdles taken to realize the entire digital functionality.

[Interviewee No. 11, Dentistry, Managing Director, Male]

## Discussion

### Principal Findings

This qualitative interview study with 19 interviewees provides new insights into the role of early career medical professionals as potential drivers of digitalization within the health care sector. By exploring their digital competencies, application experiences, and perceptions of challenges, it highlights both the opportunities and limitations of this target group. The findings offer theoretical and practical implications for medical education, health care institutions, and policymaking.

### Implications of the Findings for Research and Practice

#### Learning and Applying Digital Skills

Existing research emphasizes the lack of appropriate digital training in medical curricula [55,58]. This study confirms these findings, showing that even at progressive institutions such as UWH, students acquire digital skills mostly informally—through extracurricular activities or on-the-job learning. In this context, participants reported a clear gap between the digital demands of health care practice and the content of their former education, regardless of their year of graduation. Despite being digitally socialized and confident in the use of digital offers [31,32], students expressed frustration over the absence of a structured and practice-oriented digital education. These findings support the calls of the early career medical professionals for integrating digital competencies more and more into national frameworks such as the National Competence-Based Learning Objectives Catalogue for Medicine. At UWH, the demands of Neumann et al [55] and Sorg et al [59] also become evident: early career medical professionals seek a digital education, but this potential remains largely untapped. However, the actual conditions could also enable universities to go in new ways, setting their own digital priorities. This perspective aligns with the arguments of Khafizova et al [54] and Monteiro and Leite [75], who emphasize that universities could play a greater role in preparing students for the upcoming digital reality across various practical settings. This is particularly important because, to date, early career medical professionals appear to have developed their digital competencies primarily through informal means—such as extracurricular learning or on-the-job experiences, with an immediate application process. However, it is important to critically acknowledge that the majority of learning processes are occurring outside the traditional classroom, driven by experience and social interaction [76,77]. Nevertheless, as further research pointed out, universities could play an even more important role and make their own contribution to ensure that early career medical professionals enter the workplace digitally prepared [43,44,78]. In this context, digitalization has a transformative potential in education [79]. Consequently, in medical education, it is essential for universities to rethink their approach to teaching digital competencies and to prepare early career medical professionals for a changing job market [80,81]. In this context, universities should incorporate hands-on digital skills training that focuses not only on technical proficiency but also on how to apply these tools in real-world health care settings.

#### Potentials of Early Career Medical Professionals in Driving Digitalization Forward

This study has made evident the diverse potentials of early career medical professionals, which are likewise addressed in the existing literature.

First, the perceived relevance of recent early career medical professionals for the future of health care organizations reflects broader demographic and workforce trends. As experienced professionals retire and the “war for talent” intensifies, early-career physicians are increasingly regarded as a strategic asset [82,83]. The literature confirms this development, highlighting projected personnel shortages in the health care sector and the growing importance of attracting and retaining qualified talent—including early career medical professionals [84-86]. In light of these trends, health care organizations must adapt more deliberately to the expectations of this target group—for example, by providing the desired digital infrastructure [87-89]. Taken together, these arguments suggest that the digital expectations of recent early career medical professionals alone can serve as a catalyst for change, particularly as organizations have to respond to these demands, keeping the skilled labor shortages in mind. This finding aligns with Burnes’s perspective, which conceptualizes external stakeholder expectations as key drivers of organizational change [90]. In this context, both external pressures—such as the demographic shift—and internal resources—such as early career medical professionals’ digital readiness and intrinsic motivation—interact to position early career medical professionals as important agents of digitalization. Building on Burnes’s view, this implies that health care organizations must not only acknowledge these expectations but also actively respond by implementing targeted solutions for early career medical professionals. These may include investing in talent development programs, establishing supportive digital infrastructures, or fostering a work culture that empowers early career professionals to contribute meaningfully to digital initiatives.

Second, participants in this study emphasized that early career medical professionals possess a diverse set of professional competencies and personal attributes that position them well to contribute to the advancement of digitalization. However, these characteristics are not fixed. As Asselmann and Specht [91] and Pataki-Bittó and Kapusy [92] note, early career professionals continue to develop essential traits—such as conscientiousness, assertiveness, or team orientation—during their initial years in practice. While these studies support the notion of a developmental trajectory, they also highlight its variability, as not all early career medical professionals display the same levels of confidence, initiative, or digital affinity. It is therefore critical to avoid reductive assumptions based on generational stereotypes [93,94]. Instead, intragroup differences and individual circumstances must be acknowledged, particularly when designing support systems intended to empower recent early career medical professionals to lead digital initiatives. Here, early career medical professionals’ individual strengths must be understood in relation to both their specific work environments and their individual characteristics.

#### Challenges for Early Career Medical Professionals in Advancing Digitalization and Opportunities to Solve

This study also surfaces substantial structural, personal, and regulatory barriers that restrict the actualization of early career medical professionals in making their own contribution to the advancement of digitalization. As in previous studies by Kakale [95] or Nawroth et al [96], this study also points out a lack of formal authority, hierarchical resistance, time constraints, or a fragmented regulatory environment. These findings resonate with broader critiques of the German health care system, particularly concerning overly strict data protection frameworks and their chilling effect on digitalization and innovation [97-99]. Furthermore, participants’ references to bureaucratic overload, outdated infrastructure, and the difficulty of implementing digital solutions in analog-dominated settings align with the observations made by Wendling [100], who also emphasizes the systemic nature of these obstacles. It is therefore evident that early career medical professionals face still-documented and persistent challenges within the entire health care system, dampening their intrinsic motivation to act as key drivers of digitalization.

To address these limitations, this study also highlights potential enablers of digitalization and emerging solutions. In particular, structured mentorship, leadership support, and a receptive organizational culture are frequently identified as critical success factors [101], aligning with participants’ calls for greater top-down encouragement. Such indirectly acting measures may contribute to creating an environment that increasingly supports the digital initiatives of early career medical professionals. Moreover, consistent with the change management frameworks proposed by Glasl and Lievegoed [102] and Mizrak [103], the findings emphasize the necessity of coordinating diverse stakeholder interests. Early career medical professionals alone cannot be expected to navigate the often conflicting expectations of all different stakeholders alone. Here, effective professional leadership seems to be essential. As Burnes [90] notes, change is not a linear process, but rather a dynamic and ongoing interaction between internal capabilities and external pressures. This perspective is also reflected in the views of the study participants, who call on their organizations to manage emerging change processes systematically in order to respond professionally to evolving and changing conditions. In this context, it may be beneficial for organizations to implement structured change management approaches, as suggested by Mento et al [104], and to adopt targeted interventions at the individual, team, and organizational levels [105]. Using such established reference models may help to meet the growing demand for structured and sustainable change processes, impacting, in the end, also the opportunities for early career medical professionals in advancing digitalization in practical settings. Finally, the political dimension of digitalization cannot be overlooked. The literature highlights a central dilemma: fostering innovation while ensuring robust data protection [106,107]. This tension is also frequently emphasized by participants in this study, underscoring the need for appropriate and balanced solutions by policymakers.

#### Professional Identity Development as an Explanatory Lens

The findings can be interpreted through professional identity development, which conceptualizes early career as a phase in which early career medical professionals internalize professional norms and negotiate who they are as practitioners [108,109]. In this transition, identity is shaped through participation in workplace communities; newcomers often remain in more peripheral positions until they gain legitimacy and influence [110,111]. This helps explain why participants—despite strong digital affinity—report limited recognition, restricted influence, and that advancing digitalization is not perceived as part of their core role, leading them to prioritize integrating into existing structures and avoiding a shaping role. Early career medical professionals’ hesitation is further reinforced by hierarchical decision-making and fear of drawing attention, as well as workload and overtime constraints that crowd out discretionary innovation work. Recent research on digital implementation also suggests that professional identity threats, time pressure, workflow fit, and organizational structures shape clinicians’ willingness to engage with digital change [112]. From a professional identity development perspective, digital change agency may therefore require identity-supportive conditions: visible role models and mentoring, plus organizational legitimation (eg, protected time and formal roles) so that being a digital physician becomes a valued identity rather than an extracurricular add-on [109,113]. This aligns with participants’ emphasis on mentorship, leadership support, and receptive culture as key enablers of a new role.

Overall, it becomes evident that a diverse range of stakeholders are playing a crucial role in enabling early career medical professionals to contribute meaningfully to the advancement of digitalization in practice. Considering this, the main recommendations for action are summarized below:

1. At the educational level, medical curricula should systematically integrate comprehensive knowledge about digitalization, digital health, or concrete digital tools. In addition, practical training should equip students with essential digital skills to prepare them for the demands of modern health care.
2. At the organizational level, it is crucial to create an environment that actively supports and promotes digital initiatives of early career medical professionals. This includes establishing mentorship programs, appropriate leadership structures, and incentive systems that genuinely value and reward digital innovation. Furthermore, young professionals should be given the necessary resources, time, and autonomy to develop and implement their own digital solutions.
3. At the political level, regulatory and legal barriers hindering digital progress should be carefully considered and, if necessary and possible, further developed.

Here, without coordinated reform across policy, education, and health care governance, the transformative potential of digitally competent early career medical professionals is likely to remain underused. In sum, a multilevel approach is therefore imperative; early career medical professionals alone cannot bear the burden of driving digital initiatives without supportive structures and an appropriate ecosystem.

### Limitations

The results must be viewed with the following limitations in mind. First, the reliance on early career medical professionals limits the generalizability of the results. In this context, older health care professionals, stakeholders within the medical environment, or lecturers could also add valuable insights and perspectives to fully explore the research topic. Second, the study is based on self-reported data. In this context, the findings rely on participants’ self-reported perceptions and there is no verification of actual contributions to digitalization efforts or objective assessment of digital competencies. Therefore, participants may overstate their digital abilities and contributions. A further limitation is the absence of a systematic interrater reliability assessment and the limited extent of double-coding, which may reduce the methodological rigor and reproducibility of the coding process. Overall, the findings must be tested with a higher number of participants to generalize and test the elaborated findings, consequently following a subsequent quantitative approach. Furthermore, there is also the option of investigating other geographical areas. Here, as the research team pursues a mixed methods approach, the quantitative study is still in elaboration.

### Conclusion

To sum it up, this study revealed the perceived potential and challenges that early career medical professionals experience as they seek to advance digitalization in the health care sector. Early career medical professionals see themselves as well-positioned to drive digitalization, outlining their organizational, personal, professional, and social potential. However, they also perceive facing different organizational, personal, and professional barriers, hindering them from making an appropriate contribution to push digitalization. Consequently, early career medical professionals request support options, especially through improved digital education, organizational support, and changed regulations by the state. In conclusion, from their own perspective, early career medical professionals are an underused resource in the digitalization of health care, which is caught between high motivation and structural obstacles in the entire health care system.

## Supplementary material

10.2196/86107 Multimedia Appendix 1 Interview guide.

10.2196/86107 Checklist 1 COREQ checklist.

## Abbreviations

AI: artificial intelligence
COREQ: Consolidated Criteria for Reporting Qualitative Research
UWH: Witten/Herdecke University
