# Supplementary material for: Exploring the Role of Early Career Medical Professionals From a Digital-Oriented University in Germany in Promoting Digital Health in Professional Settings: Qualitative Interview Study
Source: JMIR Med Educ. 2026 Jun 24;12:e86107. doi: 10.2196/86107 (PMC13293368; doi:10.2196/86107)
Supplement: Multimedia Appendix 1 [file mededu-v12-e86107-s001.docx]

Interview Guide

| **Part** | **Main question** |
| --- | --- |
| **Introduction** | |
| Introduction | Please briefly describe your professional career and your current position. |
| **Learning digital skills** | |
| Learning digital skills | What professionally relevant digital skills or knowledge regarding digital health or digitalization have you acquired – during your studies, privately, through self-study, on the job or in other contexts? |
| **Practical perspective (current status, potential, challenges)** | |
| Application of digital skills | How do you use your digital skills in your professional environment? |
| Potentials of digitalization and early career medical professionals | What potential do early career medical professionals have to shape a new digital reality? Why can this target group make a positive contribution? |
| Challenges of digitalization and early career medical professionals | What prevents early career medical professionals from advancing digitalization within their practical settings? |
| **Overcoming challenges** | |
| Strategies for overcoming challenges | How can these challenges be overcome and what is needed to do so? |
| End | |
| Conclusion | Are there any other topics relevant to you that we have not yet discussed? |
