# Supplementary material for: Exploring the Role of Early Career Medical Professionals From a Digital-Oriented University in Germany in Promoting Digital Health in Professional Settings: Qualitative Interview Study
Source: JMIR Med Educ. 2026 Jun 24;12:e86107. doi: 10.2196/86107 (PMC13293368; doi:10.2196/86107)
Supplement: Checklist 1 [file mededu-v12-e86107-s002.docx]

| **Number** | **Title** | **Question** | **Answer** |
| --- | --- | --- | --- |
| **Personal Characteristics** | | | |
| 1 | Interviewer/facilitator | Which author/s conducted the interview or focus group? | CK conducted all interviews. |
| 2 | Credentials | What were the researcher’s credentials? E.g. PhD, MD | The team consists of 4 researchers from diverse backgrounds. CK has a study background in economics. JE has a study background in veterinary medicine and educational media. TSB has a study background in nursing, health sciences and applied ethics. JN has a study background in business psychology. JN and TSB have a doctoral degree in health sciences (Dr. rer. medic.). JE has a doctoral degree in veterinary medicine (Dr. med. vet.) JE, TSB and JN bring extensive experience from diverse research projects with qualitative research methods. In his master thesis, CK conducted a qualitative interview study. |
| 3 | Occupation | What was their occupation at the time of the study? | JE holds a full-time professorship for didactics and educational research in healthcare. TSB holds a full-time professorship (junior) for digital health. JN works as a post-doc for didactics and educational research in healthcare. All of them work at UW/H. CK works as an entrepreneur and as an external doctoral researcher. |
| 4 | Gender | Was the researcher male or female? | The research team consists of 2 women (TSB, JN) and 2 men (JE, CK). |
| 5 | Experience and training | What experience or training did the researcher have? | JE has been professor of didactics and educational research in healthcare since October 1, 2014 and has also been vice president of UW/H since January 1, 2017. He has extensive experience in qualitative research projects. TSB has been a junior professor since July 2024 and brings in expertise in the intersection of nursing, digital health and applied ethics. Her research focusses on acceptance and use of digital health in private and work environments as well as usability and participatory research. JN has been a post-doc since July 2024 and brings interdisciplinary expertise at the intersection of medicine, psychology, and media didactics, along with research experience on interactions in virtual learning environments, providing a strong foundation for qualitative analysis. Her background in analyzing attitudes and learning processes in digital contexts particularly equips her to interpret complex qualitative data with nuance. CK has been a student research assistant for 5 years. His study background is economics. |
| **Relationship with participants** | | | |
| 6 | Relationship established | Was a relationship established prior to study commencement? | The research team recruited some participants from their own professional network. However, there was no deeper personal or work-related relationship. The interview was not influenced by specific personal conditions. With some participants, the research team had even no personal relationship before. Before conducting the interviews, the participants were informed about the general conditions via an information letter. |
| 7 | Participant knowledge of the interviewer | What did the participants know about the researcher? e.g. personal goals, reasons for doing the research | In advance, the participants were just informed that the research project is part of CK's dissertation, supervised by TSB, JE and JN. |
| 8 | Interviewer characteristics | What characteristics were reported about the interviewer/facilitator? e.g. Bias, assumptions, reasons and interests in the research topic | The interviewers’ characteristics and intentions were not reported in detail. However, CK has reported that the research project is part of his overall doctoral project. |

Domain 2: Study Design

| **Number** | **Title** | **Question** | **Answer** |
| --- | --- | --- | --- |
| **Theoretical framework** | | | |
| 9 | Methodological orientation and Theory | What methodological orientation was stated to underpin the study? e.g. grounded theory, discourse analysis, ethnography, phenomenology, content analysis | A qualitative content analysis (Kuckartz 2018) was conducted. |
| **Participant selection** | | | |
| 10 | Sampling | How were participants selected? e.g. purposive, convenience, consecutive, snowball | The participants were selected according to three clear criteria. First, only human medical and dentistry graduates from UW/H were considered. Second, only medical graduates who have encountered digitalization in their studies or practical work were integrated. Third, only graduates from the last 15 years were included. On this basis, a list of potential participants was compiled, and interviews were conducted until theoretical saturation was reached. |
| 11 | Method of approach | How were participants approached? e.g. face-to-face, telephone, mail, email | The potential participants were approached via four channels: face-to-face, telephone, mail and social media, i.e on linkedin. After agreeing to participate in the study, further communication also took place on these four platforms. |
| 12 | Sample size | How many participants were in the study? | All in all, 19 participants (11 men; 8 women) took part in the study. |
| 13 | Non-participation | How many people refused to participate or dropped out? Reasons? | 50 Participants were invited to take part in the study via multiple channels, including face-to-face meetings, telephone interviews, email, and social media platforms. 25 participants did not responded to the invitation, and 6 dropped out due to time constraints or personal reasons. Thus, 38% of all invited people gave an interview – this corresponds to 19 participants. |
| **Setting** | | | |
| 14 | Setting of data collection | Where was the data collected? e.g. home, clinic, workplace | 18 interviews took place via the online video platform Zoom and 1 interview was conducted in person on site. Thus, most of the interviews took place online, as the barriers to participation could be lowered that way. |
| 15 | Presence of non-participants | Was anyone else present besides the participants and researchers? | In 18/19 interviews, only the participants and CK took part in the meetings. In one case, the child of a participant (<1 year) was in the same room as the participant. With the exception of a short break, this had no influence on the interview conduction. |
| 16 | Description of sample | What are the important characteristics of the sample? e.g. demographic data, date | 19 participants (11 men and 8 women) took part in the study. The youngest age of a participant is 26, the oldest 42 – the average age is 33. 17 people studied human medicine, 2 people have taken dentistry. 13 people are working in a classical medical position, i.e. as physicians, and 6 in an entrepreneurial setting, i.e. as a co-founder. |
| **Data collection** | | | |
| 17 | Interview guide | Were questions, prompts, guides provided by the authors? Was it pilot tested? | The research team developed a semi-structured interview guide along 5 focus areas and provided up to 4 questions per block. Prior to conducting the interviews, the research team successfully tested the practical applicability with a smaller pilot group of 3, afterwards finalizing the elaborated questions. |
| 18 | Repeat interviews | Were repeat interviews carried out? If yes, how many? | No repeat surveys were conducted. |
| 19 | Audio/visual recording | Did the research use audio or visual recording to collect the data? | All interviews were audio and visually recorded – twice to secure the process technically. |
| 20 | Field notes | Were field notes made during and/or after the interview or focus group? | Notes were taken both during and after the interviews, focusing on the key findings, the respective interview situation and the interview atmosphere. |
| 21 | Duration | What was the duration of the interviews or focus group? | The interviews lasted between 20 and 48 minutes. On average, each interview lasted 32 minutes. The duration varied depending on the flow of conversation and the contribution of the individual participant. |
| 22 | Data saturation | Was data saturation discussed? | As part of the joint evaluation, the research team reviewed the progress in an interim stage (after 10 interviews) and recorded open areas, i.e. still required target persons. After further 9 interviews, the research team jointly determined that theoretical saturation was reached. |
| 23 | Transcripts returned | Were transcripts returned to participants for comment and/or correction? | The participants were offered to check the transcripts for comments and correction. However, this was never utilized by anyone. |

**Domain 3: Analysis and findings**

| **Number** | **Title** | **Question** | **Answer** |
| --- | --- | --- | --- |
| **Data analysis** | | | |
| 24 | Number of data coders | How many data coders coded the data? | 2 people (CK, TSB) coded the first two interviews separately to develop the code tree. They met twice to discuss the results. Afterwards, CK coded the following 17 interviews on his own. In the following, TSB, JE and JN checked the coding and provided in several rounds critical feedback, which CK then incorporated to the satisfaction of the entire research team. |
| 25 | Description of the coding tree | Did authors provide a description of the coding tree? | The coding tree is descripted in chapter 3, “results”. |
| 26 | Derivation of themes | Were themes identified in advance or derived from the data? | 5 supper-categories and 4 subcategories were determined deductively at the beginning, 1957 codes with 1640 coded segments were then identified and assigned inductively based on the data. Thus, a deductive-inductive procedure was defined and implemented afterwards. |
| 27 | Software | What software, if applicable, was used to manage the data? | The research team used the program Sasaya_1.1.1 from RWTH Aachen for transcription and MAXQDA (Version 24) by Verbi for the following analysis. |
| 28 | Participant checking | Did participants provide feedback on the findings? | The research team presented the results to some participants. |
| **Reporting** | | | |
| 29 | Quotations presented | Were participant quotations presented to illustrate the themes / findings? Was each quotation identified? e.g. participant number | For each theme block, a participant quotation was presented to illustrate the main research results. Each quotation was identified by a specific participating number. |
| 30 | Data and findings consistent | Was there consistency between the data presented and the findings? | The data presented are showing the main findings. |
| 31 | Clarity of major themes | Were major themes clearly presented in the findings? | The presentation of the findings is clearly structured around five major themes. The results of each theme are summarized in the main text. |
| 32 | Clarity of minor themes | Is there a description of diverse cases or discussion of minor themes? | Some cases or minor themes are also addressed in the presentation of the main findings (Chapter 3). However, the reporting focus was not on the description of detailed cases. |
